# Associations of long-term fluctuation in blood pressure and ocular perfusion pressure with visual field progression in normal-tension glaucoma

**DOI:** 10.1186/s12886-024-03454-1

**Published:** 2024-05-10

**Authors:** Na Young Lee, Da Young Shin, Chan Kee Park

**Affiliations:** 1 Department of Ophthalmology, College of Medicine, The Catholic University of Korea, 505 Banpo-dong, Seocho-ku, 137-701 Seoul, Korea; 2 Department of Ophthalmology, Eunpyeong St. Mary’s Hospital, Seoul, Korea; 3 Department of Ophthalmology, Seoul St. Mary’s Hospital, Seoul, Korea

**Keywords:** Blood pressure, Normal-tension glaucoma, Ocular perfusion pressure, Progression, Visual field

## Abstract

**Backgroud:**

The aim of this study was to investigate the associations between fluctuation in blood pressure (BP), ocular perfusion pressure (OPP) and visual field (VF) progression in normal-tension glaucoma (NTG).

**Methods:**

This prospective, longitudinal study included 44 patients with NTG. Only newly diagnosed NTG patients who had not been treated with a glaucoma medication were included. Patients were examined every year for 7 years. Intraocular pressure (IOP), heart rate (HR), systolic BP (SBP), diastolic BP (DBP), ocular perfusion pressure (OPP), and diastolic ocular perfusion pressure (DOPP) were measured at the same time. Ophthalmic examinations, including perimetry, were performed also. Initial VF were compared with follow-up data after 7 years.

**Results:**

After 7 years of follow-up, 9 of the 44 patients showed VF progression. The standard deviation (SD) of SBP and OPP were significantly associated with VF progression (*P* = 0.007, < 0.001, respectively). Multiple regression analysis showed that VF progression was significantly associated with SD of OPP (odds ratio, OR = 2.012, 95% CI = 1.016–3.985; *P* = 0.045).

**Conclusions:**

Fluctuation in OPP was associated with VF progression in patients with NTG.

## Background

Intraocular pressure (IOP) is the most important causative risk factor for glaucoma [1, 2]. Previous studies have shown that lowering IOP slows visual field (VF) progression even in patients with normal-tension glaucoma (NTG) [1, 2]. However, several studies have shown that glaucoma often progressed, even when the IOP had been lowered [1, 3]. The investigators proposed that a combination of factors other than IOP was significantly associated with progression. Vascular factors have also been identified as risk factors [4–6]. 

Our previous population comprised of Korean adults revealed that patients with higher blood pressure (BP) variability developed primary open angle glaucoma significantly more frequently than did patients with lower BP variability [7]. Fluctuation of BP may trigger ischemic damage in small cerebral vessels, and repetitive peaks and troughs in BP cause endothelial dysfunction and breakdown of the blood–brain barrier [8]. Although vascular factors have been studied as risk factors in glaucoma, they are more related to the progression rather development of glaucoma [9]. 

However, to our knowledge, no previous report has evaluated the association between progression of glaucoma and long-term fluctuation of BP including ocular perfusion pressure (OPP). In the present study, we evaluated the associations of fluctuation in BP with OPP and VF progression in NTG patients.

## Methods

We conducted a single-center, prospective, longitudinal study. Newly diagnosed NTG patients who had not been treated with a glaucoma medication were recruited from the glaucoma clinic of Seoul St. Mary’s Hospital. This research was approved by the St. Mary’s Hospital Institutional Review Board and all relevant principles of the Declaration of Helsinki were followed. All eligible patients who were willing to participate signed an informed consent form approved by an institutional review board.

The inclusion criteria for this study were as follows: (1) an age of 45 to 75 years, (2) best-corrected visual acuity ≥ 20/30, (3) untreated IOP < 22 mmHg, (4) open angle on gonioscopy, (5) glaucomatous optic disc (diffuse or localized rim thinning, vertical cup-to-disc ratio > 0.6, and/or notching in the neuroretinal rim), (4) VF loss consistent with glaucoma (≥ 3 adjacent points significant at *P* < 0.05 with one of these points being significant at *P* < 0.01, or a cluster of ≥ 2 adjacent points significant at *P* < 0.01), and (6) central corneal thickness ranging from 540 to 560 μm. If both eyes met these criteria, one eye was randomly selected for analysis.

The exclusion criteria were (1) systemic diseases such as hypertension, arrhythmia or cardiovascular disease which could affect BP or heart rate (HR), (2) other ocular disease, such as corneal abnormalities or retinal disease, and (3) a history of intraocular surgery, argon laser treatment, or laser trabeculoplasty.

Newly diagnosed NTG patients were examined at baseline (without medication), and they then started to use one anti-glaucoma eyedrop. Throughout the 7 year study period, the patients only ever used one eyedrop, and no patient underwent ocular surgery or laser treatment. All participants visited the hospital every 3 or 6 months according to the routine glaucoma treatment schedule and underwent ophthalomic examination including IOP measurement, VF examination using the Swedish interactive threshold algorithm Standard 24 − 2 (Humphrey; Carl Zeiss Meditec). All participants were specifically asked to visit the hospital at 4:00 P.M on ‘the study days’ at one-year intervals and were examined in terms of IOP, HR, systolic BP (SBP), and diastolic BP (DBP). Only test results measured on ‘the study days’ (8 times in total) were used in this study. Every IOP measurement was obtained by one blinded glaucoma specialist. The IOP values at each visit were the average of three consecutive measurements obtained using Goldmann applanation tonometry. The HR, SBP and DBP of the brachial artery were measured twice in the sitting position using a standard automated BP cuff. The average value of the two measurements was used.

OPP and diastolic OPP (DOPP) were calculated using the following formulae:

$${\rm{OPP}}\,{\rm{ = }}\,\left( {{\rm{DBP}}\,{\rm{ + }}\,{\rm{ }}{\raise0.7ex\hbox{$1$} \!\mathord{\left/{\vphantom {1 3}}\right.\kern-\nulldelimiterspace}\!\lower0.7ex\hbox{$3$}}\left( {{\rm{SBP - DBP }}} \right)\,{\rm{ \times }}\,{\rm{ }}{\raise0.7ex\hbox{$2$} \!\mathord{\left/{\vphantom {2 3}}\right.\kern-\nulldelimiterspace}\!\lower0.7ex\hbox{$3$}}\,{\rm{-}}\,{\rm{ IOP}}} \right.$$

$${\rm{DOPP}}\,{\rm{ = }}\,{\rm{DBP}}\,{\rm{-}}\,{\rm{IOP}}$$

The standard deviation (SD) of SBP and DBP were calculated from the respective mean values at each visit. Fluctuations in IOP, BP, OPP, and DOPP were calculated as the SD over eight visits [7]. 

In this study, VF progression was defined using the Early Manifest Glaucoma Trial criteria [9, 12]. In the Early Manifest Glaucoma Trial, tentative VF progression was defined as three or more indicators of progression at the same location on three consecutive tests. Participants who showed tentative progression at their last visit 7 years later were reexamined 1 month thereafter to confirm VF progression. At the re-examination visit, only the VF test was performed. Patients were assigned to VF progression and non-progression groups.

The Mann-Whitney U test was used to compare the non-grogression and progression groups, because progression group is small samples (*n* = 9). To identify factors associated with progression, univariate and multivariate logistic regression analyses were performed. Variables significant at *P* < 0.05 in the univariate analysis were included in the multivariate model. We used Spearman correlation analysis to evaluate the relationships between VF progression and other variables. *P* values < 0.05 were considered to indicate statistical significance. All statistical analyses were performed using SPSS for Windows software (ver. 18.0; SPSS Inc., Chicago, IL, USA).

## Results

In total, 44 patients with NTG were enrolled in the study. Table 1 summarizes the demographic and clinical characteristics of the participants. Of the 44 patients, 9 demonstrated progression. Age, gender, IOP, refraction, baseline mean deviation, pattern SD, central corneal thickness, SBP, DBP, OPP, and DOPP showed no significant differences between the groups (Table 2). However, the visit-to-visit variability in SBP and OPP (defined on the basis of the SDs) differed significantly between the patients with and without progression ( *P* = 0.007 and *P* < 0.001 respectively; Table 2). The relationships between risk factors and VF progression were evaluated by logistic regression analysis. In the univariate analysis, progression was associated with the SD of SBP (odds ratio [OR] = 1.239; 95% confidence interval [CI] = 1.036–1.482; *P* = 0.019) and the SD of OPP (OR = 2.165; 95% CI = 1.205–3.891; *P* = 0.010; Table 3). In a subsequent multiple regression analysis, including progression as the dependent parameter and variables with *P* values < 0.05 in the univariate analysis as independent variables, progression was significantly associated with the SD of OPP (OR = 2.012, 95% CI = 1.016–3.985; *P* = 0.045; Table 3).

**Table 1:** Baseline ocular characteristics, blood pressure (BP), ocular perfusion pressure (OPP) and diastolic ocular perfusion pressure (DOPP) in patients with normal-tension glaucoma (mean ± standard deviation)

Characteristics	Baseline	1yr	2yr	3yr	4yr	5yr	6yr	Last visit
Sex (M/F)	11/33							
Age (y)	59.91 ± 7.12							
Refraction (D)	-0.88 ± 2.53							
CCT (µm)	544.6 ± 13.6							
MD (dB)	-3.54 ± 2.73							-3.67 ± 3.13
PSD (dB)	4.72 ± 3.78							4.80 ± 3.57
IOP (mmHg)	15.84 ± 2.90	15.52 ± 2.41	14.57 ± 2.80	14.86 ± 2.57	15.84 ± 2.39	15.61 ± 2.39	15.77 ± 2.34	15.42 ± 1.93
HR (n)	74.57 ± 9.50	72.55 ± 8.72	72.55 ± 8.72	70.30 ± 8.48	74.73 ± 10.77	74.55 ± 12.14	72.45 ± 7.91	73.01 ± 8.00
SBP (mmHg)	128.89 ± 14.91	123.74 ± 14.32	120.14 ± 14.95	119.30 ± 13.24	121.91 ± 13.26	123.68 ± 14.87	123.14 ± 14.79	122.84 ± 11.65
DBP (mmHg)	79.75 ± 8.67	77.52 ± 10.04	75.45 ± 7.50	79.16 ± 8.35	77.73 ± 9.82	77.48 ± 8.06	77.77 ± 9.05	77.89 ± 6.84
OPP (mmHg)	47.76 ± 6.97	46.13 ± 6.93	46.67 ± 8.37	45.88 ± 6.84	47.12 ± 6.28	46.71 ± 7.22	46.15 ± 6.65	40.46 ± 5.84
DOPP (mmHg)	63.32 ± 8.61	61.24 ± 10.12	63.20 ± 9.81	60.59 ± 8.09	63.91 ± 8.32	62.11 ± 9.83	61.70 ± 8.71	62.47 ± 6.98

M, male; F, female; y,year; CCT, central corneal thickness; MD, mean deviation; PSD, pattern standard deviation; SBP, systolic blood pressure; DBP, diastolic blood pressure; SD, standard deviation; Continuous data are mean $\pm$ mean standard deviation unless otherwise indicated

**Table 2:** Comparison of baseline ocular characteristics, blood pressure (BP), ocular perfusion pressure (OPP) and diastolic ocular perfusion pressure (DOPP) in patients with normal-tension glaucoma (mean ± standard deviation)

Factor	Non progression group	Progression group	*p*-value
No. of cases	35	9	
Age (y)	60.71 ± 7.50	57.11 ± 4.76	0.091
Sex (M/F)	8/27	3/6	0.647
CCT (µ)	543.6 ± 14.4	548.3 ± 9.6	0.373
Refraction (diopter)	-1.12 ± 2.78	0.08 ± 0.48	0.208
Baseline IOP (mmHg)	15.71 ± 2.84	16.33 ± 3.24	0.574
Mean IOP (mmHg)	15.91 ± 1.79	15.83 ± 2.48	0.474
Baseline MD (dB)	-3.92 ± 2.90	-2.07 ± 1.20	0.070
Baseline PSD (dB)	4.86 ± 4.13	3.92 ± 1.51	0.282
Baseline HR (n)	74.74 ± 9.13	73.89 ± 11.41	0.813
Baseline SBP (mmHg)	124.8 ± 13.4	119.6 ± 17.5	0.405
Baseline DBP (mmHg)	78.5 ± 9.	73.7 ± 10.8	0.140
Baseline OPP (mmHg)	46.7 ± 6.2	43.5 ± 9.3	0.261
Baseline DOPP (mmHg)	62.0 ± 9.6	57.9 ± 11.6	0.226
Mean SBP (mmHg)	123.51 ± 11.70	120.24 ± 11.73	0.459
Mean DBP (mmHg)	78.00 ± 7.07	77.46 ± 6.23	0.836
Mean OPP (mmHg)	40.55 ± 5.81	40.10 ± 6.31	0.689
Mean DOPP (mmHg)	62.63 ± 7.36	61.63 ± 7.36	0.842
SD of IOP (mmHg)	1.76 ± 0.68	1.98 ± 0.80	0.416
SD of SBP (mmHg)	7.46 ± 4.12	12.05 ± 4.97	0.007^*^
SD of DBP (mmHg)	5.22 ± 2.24	7.67 ± 4.36	0.023^*^
SD of OPP (mmHg)	3.51 ± 1.53	6.11 ± 2.49	< 0.001^*^
SD of DOPP (mmHg)	5.61 ± 2.49	5.60 ± 2.89	0.991

M, male; F, female; y,year; CCT, central corneal thickness; MD, mean deviation; PSD, pattern standard deviation; SBP, systolic blood pressure; DBP, diastolic blood pressure; Continuous data are mean $\pm$ mean standard deviation unless otherwise indicated

*: Statistical significance (*p* < 0.05)

**Table 3:** Clinical characteristics associated with visual field progression in patients with normal-tension glaucoma by univariate and multivariate regression analyses. CCT, central corneal thickness;; IOP, intraocular pressure; MD, mean deviation; PSD, pattern standard deviation; HR, heart rate; SBP, systolic blood pressure; DBP, diastolic blood pressure; OPP, ocular perfusion pressure; DOPP, diastolic ocular perfusion pressure; SD, standard deviation

Variable	Univariate analysis		Multivariate analysis	
	Odds ratio, 95% CI	*p* value	Odds ratio, 95% CI	*p* value
Age	0.926, 0.827–1.036	0.181		
Female gender (vs. male)	0.593, 0.120–2.920	0.520		
Refraction	1.560, 0.716–3.396	0.263		
CCT	1.028, 0.970–1.089	0.349		
Baseline IOP	1.080, 0.831–1.403	0.656		
Baseline MD	1.504, 0.929–2.435	0.097		
Baseline PSD	0.926, 0.740–1.158	0.502		
Baseline HR	0.990, 0.915–1.072	0.808		
Baseline SBP	0.990, 0.942–1.040	0.686		
Baseline DBP	1.005, 0.920–1.098	0.907		
Baseline OPP	0.978, 0.875–1.094	0.702		
Baseline DOPP	0.996, 0.914–1.086	0.935		
Mean IOP	1.154, 0.785–1.696	0.465		
Mean HR	1.023, 0.926–1.129	0.658		
Mean SBP	0.976, 0.917–1.039	0.451		
Mean DBP	0.988, 0.887–1.101	0.832		
Mean OPP	0.987, 0.869–1.120	0.837		
Mean DOPP	0.978, 0.879–1.088	0.681		
SD of IOP	1.585, 0.533–4.720	0.408		
SD of SBP	1.239, 1.036–1.482	0.019^*^	1.048, 0.834-1.318	0.687
SD of DBP	1.296, 0.990–1.698	0.059		
SD of OPP	2.165, 1.205–3.891	0.010^*^	2.012, 1.016–3.985	0.045^*^
SD of DOPP	0.998, 0.745–1.337	0.990		

^*^: Statistical significance (*p* < 0.05)

The relationship between the variables and VF progression were analyzed using Spearman correlation analysis. VF progression correlated significantly with the SD of SBP (ρ = 0.404, *P* = 0.007), SD of DBP (ρ = 0.300, *P* = 0.048), and SD of OPP (ρ = 0.522, *P* < 0.001). However, no significant correlation was detected between VF progression and gender, age, IOP, mean deviation, pattern SD, SBP, DBP, OPP, DOPP, the SD of IOP, or the SD of DOPP (Table 4).

**Table 4:** The correlations of visual field progression with baseline characteristics and clinical parameters in patients with normal-tension glaucoma

Variables	ρ	*p*-value
Sex	-0.980	0.529
Age	-0.276	0.179
Refraction	0.194	0.208
CCT	0.143	0.356
Baseline IOP	0.107	0.489
Mean IOP	0.087	0.576
Baseline MD	0.276	0.070
Baseline PSD	-0.102	0.508
Baseline HR	-0.100	0.518
Baseline SBP	0.129	0.404
Baseline DBP	-0.227	0.139
Baseline OPP	-0.246	0.107
Baseline DOPP	-0.189	0.219
Mean HR	0.038	0.808
Mean SBP	-0.149	0.335
Mean DBP	-0.071	0.647
Mean OPP	0.038	0.808
Mean DOPP	-0.060	0.699
SD of IOP	0.126	0.416
SD of SBP	0.404	0.007^*^
SD of DBP	0.300	0.048^*^
SD of OPP	0.522	< 0.001^*^
SD of DOPP	-0.002	0.991

CCT, central corneal thickness; IOP, intraocular pressure; MD, mean deviation; PSD, pattern standard deviation; HR, heart rate; SBP, systolic blood pressure; DBP, diastolic blood pressure; OPP, ocular perfusion pressure; DOPP, diastolic ocular perfusion pressure; SD, standard deviation; ρ, Spearman correlation coefficient

^*^: Statistical significance (*p* < 0.05)

## Discussion

Elevated IOP is considered a major risk factor for glaucoma, and several vascular risk factors have also been identified [4, 13, 14]. Such vascular factors can lead to hypoperfusion of the optic disc and may contributes significantly to the progression of glaucoma [15–20]. 

Long-term repetitive fluctuation of BP may damage the vasculature [8]. Impaired vascular autoregulation affects the stability of ocular perfusion, and repeated ischemic/reperfusion injury triggers glaucomatous optic neuropathy [17, 21, 22]. 

Our previous study showed that patients with greater long-term BP variability developed primary open angle glaucoma significantly more frequently than did patients less variability (*P* < 0.001) in a large population-based cohort [7]. In the current study, long-term fluctuation of SBP and OPP were significantly associated with VF progression (*P* = 0.007 and *P* < 0.001, respectively). Multiple regression analysis showed that VF progression was significantly associated with the SD of OPP (OR = 2.012, 95% CI = 1.016–3.985; *P* = 0.045). In conclusion, BP fluctuation was associated with the progression of glaucoma in both studies.

Previously, Sung et al. showed that patients with NTG in the highest tertile of mean OPP fluctuation over 24 h were at greater risk of progressive VF loss than patients in the lowest fluctuation tertile [23]. They showed that short-term fluctuation of mean OPP was associated with VF progression in NTG patients. We measured visit-to-visit BP and showed that long-term fluctuation of OPP was associated with VF progression in patients with NTG. To our knowledge, this is the first report on a relationship between VF progression and long-term fluctuation of OPP in NTG patients.

A limitation of this prospective, longitudinal study should be acknowledged: the multivariate analysis had a relatively small sample size. However included participants who underwent full ophthalmic and systemic evaluation and checked BP at the same time during the follow-up period. In addition, the participants did not develop new systemic diseases and did not take systemic drug during the total observation period, which enhances the reliability of the study.

In summary, we found that long-term fluctuation of OPP was associated with VF progression in patients with NTG. Currently, interest in glaucoma treatment concerns not only IOP lowering but also vascular factors. This study highlights the risk of cardiovascular instability in association with glaucoma progression and suggests another way to manage glaucoma. Further studies are needed to more comprehensively investigate the role of vascular factors in VF progression in NTG patients.

## Data Availability

The datasets used and analysed during the current study are available from the corresponding author on reasonable request.
